# Social-cognitive correlates of risky adolescent cycling behavior

**DOI:** 10.1186/1471-2458-10-408

**Published:** 2010-07-12

**Authors:** Hans Feenstra, Robert AC Ruiter, Gerjo Kok

**Affiliations:** 1 Work and Social Psychology, Faculty of Psychology and Neuroscience, Maastricht University, Maastricht, the Netherlands

## Abstract

**Background:**

Bicycle use entails high safety and health risks especially for adolescents. Most safety education programs aimed at adolescents focus on accident statistics and risk perceptions. This paper proposes the investigation of the social-cognitive correlates of risky cycling behaviors of adolescents prior to developing safety education programs.

**Method:**

Secondary school students aged 13 to 18 years (n = 1446) filled out questionnaires regarding bicycle behavior, risky intentions, accident experience, and social-cognitive determinants as suggested by the theory of planned behavior.

**Results:**

Regression analysis revealed that the proximal variables (i.e., self-efficacy, attitudes towards drunk driving, personal norm regarding safekeeping of self and others, and compared risk) were able to predict 17% of the variance of risky behavior and 23% of the variance of risky intentions. The full model explained respectively 29% and 37% of the variance in risky behavior and risky intentions. Adolescents with positive attitudes towards risky behavior and low sense of responsibility report risky behavior, even when having been (close to) an accident.

**Conclusions:**

Adolescents realize whether they are risk takers or not. This implies that the focus of education programs should not be on risk perceptions, but on decreasing positive attitudes towards alcohol in traffic and increasing sense of responsibility instead. Cognitions regarding near accidents should be studied, the role of safe cycling self-efficacy is unclear.

## Background

The present study was set up to investigate the social-cognitive correlates of risky cycling behaviors of adolescents. Bicycles are a common means of transportation for adolescents in the Netherlands, as well as in other European countries. However, their use also entails high safety and health risks as observed in accident statistics. In 2007, over 3000 adolescents (age 16 - 24) were hospitalized and 169 died in traffic accidents [1]. In order to decrease the risk many traffic education programs have been adopted. However, most programs lack a decent empirical basis. These programs are based on accident statistics only and not on social psychological determinants of teenage cycling behavior. An insight in the social psychological determinants of teenage cycling behavior is important when behavior change is the aim of the program [2]. Interventions to promote safer cycling in adolescents should start with an assessment aiming to identify specific behaviors contributing to the health and safety problem at hand and their social-cognitive determinants. Following the formulation of program objectives, methods for change are selected that target the identified social-cognitive determinants. These methods are then translated in specific strategies that fit the intervention context and integrated into a comprehensive intervention program while anticipating program implementation and evaluation [2,3]. The present study aimed to identify relevant social-cognitive correlates of risky cycling behavior in adolescents to inform future intervention programs.

Many explanations have been put forward explaining why adolescents show more risky behaviors in general and specifically in traffic [for overviews, see [4-7]]. For instance, when children reach adolescence, this coincides with an increase in independence. Because adolescents may explore boundaries, may fail to recognize potentially harmful situations or may actually seek out risky situations, chances of encountering these situations increase, which would not happen (or happen less) under parental supervision [4]. The early adolescent period is characterized by a decrease in parental supervision [5]. Biologically, the adolescent period comes with an onset of hormones which lead to sensitivity for social approval and a tendency to show bravery in the eyes of peers. Moreover, there is an increase in exploratory and reward-seeking activities in adolescence [4]. Besides, because adolescents do have the skills to ride a bicycle safely it is often assumed that it is the adolescents' conscious decision to take risks in traffic. But is that really the case, or are there other purposes for their behavior, like 'being cool' [6]?

Reyna and Farley [7] provide an overview of explanations why adolescents may seek out situations with potential risks. For instance, they state that adolescents are capable of rational decision making but they are also, more than adults, willing to explore risky options. Whereas adults are generally risk avoidant, adolescents are likely to weigh the pros and cons of any given situation. Often the pros will outweigh the cons, because traffic is objectively quite safe and adolescents typically prefer short-term benefits over long-term benefits [7].

There is a good chance risky behavior of car drivers has its origin in the driver's younger years. Reason and colleagues suggest that people learn to act dangerously in traffic because risky behavior is often not punished, but rather perceived as advantageous [8]. Thus risky behaviors are likely to become a habitual part of one's driving style. It is therefore important to promote risk-avoiding behavior before people start driving cars and preferably during early adolescence.

Shope and Bingham [6] list a series of possible determinants explaining why young drivers run more risk than adult drivers: characteristics of the behavior (i.e. staying up late in the weekends, which leads to sleep deprivation), abilities (i.e. lack of expertise), developmental factors (i.e. brain development), behavioral factors (i.e. aggression), personality (i.e. hostility), demographics (i.e. less parental supervision), social environmental factors (i.e. peers), and physical environment (i.e. distractions). Males [9] states that the financial situation of adolescents may play a part - adolescents usually have less money to spend than adults, and consequently are forced to buy cars of lesser quality. Keating and Halpern-Felsher [5] suggest that developmental factors are the most relevant and that expertise comes with experience and practice. They state that there is no evidence that young drivers underestimate risks more than adult drivers. Reyna and Farley [7] also stress that adolescents, despite conventional wisdom, do feel vulnerable and generally overestimate risks. Indeed, after the age of 14, it can be assumed that there are no differences between teens and adults concerning the perception of risk [10]. Traffic education should therefore not focus on accuracy of risk perceptions, or on deliberately weighing pros and cons, but should promote risk-avoiding behaviors instead [7]. In addition, all these authors urge for a better understanding of the social-cognitive determinants of adolescent road use behaviors, since through those determinants behavior might be changed.

In the present study we focus on risky adolescent cycling behavior in the Netherlands from a social psychological perspective. The goal of this study is to analyze the relation between risky behavior and relevant social-cognitive determinants. The determinants measured in this study were selected based on current theoretical insights [2], specific social cognition models of human risk behavior, in particular Theory of Planned Behavior [11,12], and on expected associations with safe or unsafe cycling: risk-perceptions, attitudes, responsibility, experience with accidents, and self-efficacy.

While many causes of risky cycling behavior are known, a need for a better insight in social cognitive determinants still exists. Without a decent understanding of the social cognitive determinants underlying risky cycling behavior, education initiatives focused on behavior change are bound to fail. Accurate insights will lead to proper focal points of interventions, which increase the chance of interventions being successful in improving safer traffic behavior and reducing accidents. This study aims to contribute to a better insight in these social cognitive determinants.

## Methods

### Participants and Procedure

Data were collected among 1749 secondary school students aged 13 to 18 years from seven schools in the province of Limburg, the Netherlands, who were identified as bicyclists (i.e., they indicated to ride their bike more than three times a week). The study was approved by the Ethical Committee Psychology of the School of Psychology and Neuroscience, Maastricht University. Students from three levels of secondary education participated in the study (i.e. lower and higher general secondary education, and pre-university college). They filled out a questionnaire with self-report measures of risky cycling behavior and items measuring attitude, self-efficacy, risk judgments, intentions, and personal experiences. It took about twenty minutes to fill out the questionnaire. Questionnaires were handed out in class, where a teacher supervised the process and, if necessary, clarified any problems regarding the contents of the questionnaire.

Participants who failed to enter their name, age, sex, or any of the key measures were excluded from the analysis (*n *= 303), which resulted in a final sample of 1446 students. T-tests revealed no significant differences between excluded and included participants on age, sex, and the outcome of intention and behavior (*p*'s > .05). In the final sample 291 students (141 girls) attended lower general secondary education (20.1% of total), 569 students (302 girls) attended higher general secondary education (39.4% of total), and 277 girls and 247 boys attended pre-university college (36.2% of total). The level of education of 41 girls and 21 boys could not be established for certain (4.3% of total), but they were retained for analysis. Mean age was 15.0 (*SD *= .79) for girls as well as for boys (*SD *= .83).

### Measures

For each measure, scores on separate items that showed sufficient internal consistency (Cronbach's alpha [α] < .60) were averaged into one single index (unless otherwise indicated). Higher scores reflect a stronger presence of the concerned variable.

#### Risky cycling intention

Intention to perform dangerous cycling behavior was measured by a combination of three questions reflecting Reason's [8] subdivision of errors: The first question *"How often in the next month do you intend to break traffic rules?" *pertains to *violations *(a deviation from what is deemed safe), the second *"How often in the next month do you expect to get in a potentially harmful situation because of an error you make in traffic?" pertains *to *mistakes *(conscious but wrong decisions), and the third *"How often in the next month do you expect to break traffic rules unknowingly?" *to *slips and lapses *(unconscious errors). Scores ranged from 1 = *never *to 6 = *always *(α = .60).

#### Risky cycling behavior

The Dutch Institute for Traffic Safety Research (SWOV) has developed a questionnaire that measures risky bicycle behavior [13]. Participants were asked to state the number of times they performed 22 different kinds of intended or unintended dangerous cycling behavior in the past month (e.g., "Riding a bike when under the influence of alcohol/marijuana", "Using a cell phone whilst cycling", "Forgetting to signal when changing directions", and "Riding at night without working head/tail light"). Scores on these items ranged from 1 = *never *to 6 = *always *(α = .88).

#### Self-efficacy

Self-efficacy concerning traffic skills was measured using a comparison to other cyclists of similar age and sex regarding five issues: Controlling your bicycle, applying traffic rules, traffic situation insight, ability to withstand temptations to take risks, and ability to withstand peer pressure. Response options ranged from 1 = *much worse *to 5 = *much better *(α = .66).

#### Risk comparison

Participants were asked about their comparative risk to get a traffic accident with a single item: "Compared to other bicycle riders of my age and sex my risk of getting a traffic accident is... ". The response options ranged from 1 = *much smaller *to 5 = *much higher*.

#### Attitude towards traffic violations

Attitude toward violating traffic rules was measured using five items, e.g., "It should be up to me whether I obey the traffic rules or not", "With no traffic in sight, stopping in front of a red light makes no sense". Response options ranged from 1 = *totally disagree *to 5 = *totally agree *(α = .67).

#### Attitude towards alcohol use in traffic

Attitude towards drunk driving was measured using four items, e.g., "If someone is half-drunk, I don't mind him riding a bike", "Everyone taking part in traffic has to be sober". Response options ranged from 1 = *totally disagree *to 5 = *totally agree *(α = .78).

#### Personal norm: safety for self

Personal norm regarding one's own safety was measured using two items: "I believe I should behave myself in traffic and not only when there's cops around", "I think it's important not to endanger myself". Response options ranged from 1 = *disagree *to 5 = *agree *(Pearson's *r *= .51, *p *< .001).

#### Personal norm: safety for others

Personal norm regarding other people's safety was measured using six items, e.g., "Everyone knows that participating in traffic is risky. If someone gets hurt because of me, too bad", "I would feel terrible if someone would get hurt because of me". Response options ranged from 1 = *disagree *to 5 = *agree *(*α *= .64).

#### Perceived risk taking

Risk taking was measured using three items: "How much risk do you take in traffic as a cyclist on your own?", "how much risk do you take in traffic as a cyclist in a group of friends?", "how much risk do you take in traffic as a pedestrian?". Response options ranged from 1 = *I don't take risks *to 5 = *quite a lot of risk *(α = .71).

#### Personal experience with accidents

Two items measured participants' own experience with accidents: "Did you have an accident in the past two years so severe that you had to visit a doctor or hospital?"; response options were 1 = *no*, 2 = *nothing serious*, 3 = *had to see a doctor*, and 4 = *went to hospital*; "Did you have an accident in the past two years in which you only had material damage?"; ranging from 1 = *no *to 4 = *more than twice*. The scores on these items were combined to form one index of personal experience (Pearson's *r *= .31, *p *< .001).

#### Near accidents

One question measured near accidents: "How often did you almost have an accident", with response options ranging from 1 = *practically never *to 4 = *practically every week*.

## Results

### Risky cycling behavior and intentions

Means and standard deviations of the social-cognitive variables, intentions and behavior are presented in Table 1. Correlation analysis was used to determine bivariate (inter)relationships of the social-cognitive variables with self-report measures of dangerous cycling behavior as well as intentions to perform dangerous behavior in the next month (see Table 1). Only those variables with correlations > .05 (= *p *<.05) with behavior or intention were selected in a multivariate regression to determine the amount of explained variance in behavior.

**Table 1:** Correlation coefficients, means and standard deviations of risk behavior, risk intentions, and determinants (n = 1446)

		1	2	3	4	5	6	7	8	9	10	11	12	13	14
1	Risky intentions														
2	Risky cycling behavior	.48													
3	Safe cycling self-efficacy	-.12	-.09												
4	Attitude towards alcohol use in traffic	.29	.31	-.14											
5	Personal norm: safety self	-.42	-.34	.19	-.44										
6	Personal norm: safety others	-.33	-.29	.08	-.39	.51									
7	Age	-.03	.03	.04	.15	.01	.04								
8	Sex	.10	.12	.17	.10	-.16	-.26	.02							
9	School type*	.03	-.16	-.03	-.06	.04	.06	-.23	-.03						
10	Personal experience with accidents	.22	.19	-.06	.09	-.14	-.13	.02	-.02	.07					
11	Near accidents	.32	.25	-.09	.15	-.23	-.20	-.01	.00	.03	.36				
12	Perceived risk taking	.42	.34	-.11	.28	-.44	-.34	-.02	-.01	.15	.11	.25			
13	Attitude towards traffic violations	-.06	-.04	.04	-.01	.04	.05	.02	-.09	.03	-.07	-.04	-.02		
14	Risk comparison	-.26	-.20	.25	-.14	.26	.22	.02	.02	.00	-.17	-.23	-.23	.01	
															
	Mean	1.77	1.96	3.44	2.40	3.84	3.54	15.0	.47	Na	1.37	1.49	2.20	3.10	3.40
	SD	.67	.63	.55	.81	.69	.54	.81	.50		.58	.69	.68	.79	.84

*r *> |.05| has *p *< .05; *r *> |.07| has *p *< .01; *r *> |.09| has *p *< .001.

* n = 1384.

### Predicting risky cycling behaviors

A regression analysis was run using the Enter method, where the variables correlating (*r*'s > .09; *p *< .001) with the behavior scale were entered in four blocks (Table 2). In the first block the so-called proximal variables (i.e., self-efficacy, attitudes, and norms) were entered. These proximal variables were able to explain 17% of the total variance in risk behavior. In the second block past experience with (near) accidents was added, which lead to an increase of 2% in explained variance. In the third block sex was added (an increase of 1% in explained variance), and in the final block perceived risk taking and intention. The full model explained 29% of the total variance in risky cycling behavior.

**Table 2:** Regression analyses: Risky cycling behavior, intention, and determinants (n = 1446)

	Risky cycling behavior					Risky cycling intention				
		**Model**					**Model**			
		**1**	**2**	**3**	**4**		**1**	**2**	**3**	**4**

	***r***	**B**	**B**	**B**	**B**	***r***	**B**	**B**	**B**	**B**

Self-Efficacy	-.09^***^	.00	.00	-.01	-.01	-.12^***^	-.01	-.01	-.01	.00
Risk comparison	-.20^***^	-.11^***^	-.07^**^	-.07^**^	-.03	-.26^***^	-15^***^	-11^***^	-11^***^	-07^**^
Attitude towards alcohol use in traffic	.31^***^	.17^***^	.17^***^	.17^***^	.13^***^	.30^***^	.11^***^	.10^***^	.10^***^	.04
Attitude towards traffic violations						-.06^*^	-.04	-.03	-.03	-.02
Personal norm: safety self	-.34^***^	-.18^***^	-.16^***^	-.15^***^	-.04	-.42^***^	-.28^***^	-.25^***^	-.25^***^	-.16^***^
Personal norm: safety others	-.29^***^	-.11^***^	-.10^**^	-.08^**^	-.04	-.33^***^	-.11^***^	-.09^**^	-.09^**^	-.05
Past accident involvement	.19^***^		.09^**^	.08^**^	.06^*^	.22^***^		.08^**^	.08^**^	.06^*^
Near accidents	.25^***^		.12	.13^***^	.06^*^	.32^***^		.17^***^	.17^***^	.11^***^
Sex	.12^***^			.05^*^	.04	.10^***^			.01	-.01
Perceived risk taking	.34^***^				.10^***^	.42^***^				.17^***^
Intention	.48^***^				.32^***^					
Unsafe behavior						.48^***^				.29
										
R2		.17	.19	.20	.29		.23	.27	.27	.37

^*^p < .05; ^**^p < .01; ^***^p < .001

### Predicting risky cycling intentions

A regression analysis was run using the Enter method, where all variables correlating with the intention scale were entered in four blocks (Table 2). The same configuration was used as before with the behavior scale. The proximal variables were able to explain 23% of the total variance in intention. Adding past experience with (near) accidents to the model led to an increase of 4% in explained variance. Sex did not increase the amount of explained variance any further. The addition of perceived risk taking and risky cycling behavior led to 37% of the total variance in intention to be explained by the full model.

To correct for the influence of the different schools, the data was also analysed using hierarchical linear modelling with school as random effect variable. These analyses yielded identical findings. The amount of variance in the outcome variables explained by school membership was less than 1%.

## Discussion

The object of this article was to identify relevant social-cognitive correlates of unsafe cycling behaviors. In this study ten determinants of behavior and intention were identified (i.e., sex, self-efficacy, risk comparison, attitude toward alcohol in traffic, personal norm towards not endangering one's self, personal norm towards not endangering others, past accident involvement, near accident involvement, perceived risk taking, and intention to behave risky for behavior and vice versa). More specifically, the object of this study was to identify those social-cognitive correlates that are useful for interventions aiming to change behavior. Since the variables in the three latter blocks are either unchangeable (sex, prior experience), practically similar to the dependent variable (perceived risk taking), or measured simultaneously (intention), the focus regarding the results should be on the proximal variables (i.e. self-efficacy, risk comparison, attitude towards alcohol in traffic and the personal norms). These five variables were able to predict 17% of the variance in unsafe adolescent cycling behavior and 23% of the variance in risky cycling intentions.

The measures of attitudes, norms, and self-efficacy were correlated with intentions and behavior in an unsurprising way. Self-efficacy towards safe cycling skills was negatively correlated with risky cycling behavior and risky cycling intentions. Participants scoring high on personal norm to keeping one's self and others safe scored lower on risky cycling behavior and risky cycling intentions. Having a positive attitude towards being under the influence of alcohol related to higher scores on both risky cycling behavior and risky cycling intentions. However, the two variables measuring experience with accidents and near accidents were both positively associated with more risk taking. This positive association could mean two things. First, adolescents with risky cycling styles may encounter more dangerous situations and may therefore encounter more accidents and near accidents. Second, adolescents who report having an accident or near accidents in the past two years report dangerous cycling behavior during the past month. The latter explanation suggests that adolescents do not automatically learn from (near) accidents and thus do not change their risky behavior based on previous experiences, which is in line with Reyna and Farley [7] Finally, from the regression analysis we could conclude that adolescents taking more risks in traffic (or intending to) see themselves more as risk takers, care less about their own safety and that of others, and are more tolerant of drunken driving.

The present study has some limitations. First and foremost, the variables used to predict behavior were not measured in the best possible way [14], namely on the same level as the behavior (cf. correspondence principle [15]). At the start of this study little was known about specific risky behaviors. Essential knowledge on the relation between cycling behaviors and accident involvement is still lacking. In order to promote safer cycling we must know more about this relationship. However, adequate epidemiological studies into this relation are very complex [16] and are currently unavailable. Besides, because questionnaires have to be short in order to guarantee completion by adolescents, it was impossible to create items for variables like self-efficacy and personal norm corresponding to every single risky behavior. Second, this study did not systematically explore all potentially relevant social-cognitive determinants from current behavior models, such as the Social Cognitive Theory [17]. For instance, social influence of peers might also be a determinant of adolescents' risky traffic behavior [18]. Future studies should also include other potentially relevant variables, i.e. automatic behavior or habits [19], subjective social norm, and descriptive norm. Furthermore, intention was not measured according to Theory of Planned Behavior. Rather, it was a combination of three questions reflecting Reason's [8] subdivision of errors. The reliability of intention was quite low, which raises questions about its validity. Nevertheless, the full model explained 37% of the variance in this measure of intention. Finally, behavior should ideally be measured at a later moment in time than the determinants to strengthen the causal interpretation of the associations between determinants and behavior.

## Conclusions

In conclusion, the social-cognitive determinants measured in this study were moderately effective in predicting risky cycling behaviors measured with the self-report questionnaire. It is striking that adolescents' experience with accidents apparently does not promote safer traffic behavior. As mentioned in the introduction, this may be a reflection of the developmental stage adolescents are in. Adolescents are aware of their risk taking behavior and it seems evident that interventions to promote safer cycling should therefore not focus on risk perceptions. There is one possible exception as it might be useful to pay attention to susceptibility of accident involvement in relation to experience with (near) accidents. Near accidents occur more often than actual accidents. On the one hand adolescents might therefore learn that potential accidents usually have a positive ending [20]. On the other hand, they might learn that they lack of control over getting an accident, in which case some sort of helplessness is displayed. The corresponding cognition might be "it doesn't matter how I behave, I cannot control the occurrence of an accident". In that case self-efficacy towards safe cycling should be improved.

The focus of traffic education programs should thus be more on promoting traffic expertise (especially at an earlier age), acceptance of responsibility, self-efficacy (to increase the notion of control over their own behavior in relation to accident involvement), and probably resistance to social pressure [21], instead of on risk perception and fear. However, even if one would never display risky behavior in traffic, this can not diminish the risk of getting an accident. In traffic, one is almost never alone and, except for 'one-sided accidents in which no other party is involved, almost always dependent on other traffic participants. Further studies on adolescents cycling should target other potentially relevant determinants, the prediction of future behavior, and the relationship between questionnaire measures of behavior and actual accident involvement. Furthermore, the cognitions of adolescents regarding near accidents should be studied. Near accidents may provide an opportunity for traffic education, because practically every traffic participant has a recollection of a 'close call'. It is important to know how people deal with these situations before interventions can be attuned to them. Taking all of the above into account, and following similar approaches in other domains of health promotion [22-24], it should be possible to create safety interventions tailored to the needs of the target population.

## Competing interests

The authors declare that they have no competing interests.

## Authors' contributions

HF carried out the cross-sectional survey, the procedures for data cleaning, reduction and analysis, and drafted the manuscript. RR participated in the design of the study, supervised the study conduct and the data analyses and helped to draft the manuscript. GK participated in the design of the study, supervised the study conduct and the data analyses and helped to draft the manuscript. All authors read and approved the final manuscript.

## Pre-publication history

The pre-publication history for this paper can be accessed here:

http://www.biomedcentral.com/1471-2458/10/408/prepub
